# Overexpression of *Cinnamoyl-CoA Reductase 2* in *Brassica napus* Increases Resistance to *Sclerotinia sclerotiorum* by Affecting Lignin Biosynthesis

**DOI:** 10.3389/fpls.2021.732733

**Published:** 2021-09-23

**Authors:** Dongxiao Liu, Jian Wu, Li Lin, Panpan Li, Saifen Li, Yue Wang, Jian Li, Qinfu Sun, Jiansheng Liang, Youping Wang

**Affiliations:** ^1^Key Laboratory of Plant Functional Genomics of the Ministry of Education, Yangzhou University, Yangzhou, China; ^2^Jiangsu Key Laboratory of Crop Genomics and Molecular Breeding, Yangzhou University, Yangzhou, China; ^3^Department of Biology, School of Life Sciences, Southern University of Science and Technology, Shenzhen, China

**Keywords:** *Brassica napus*, *Sclerotinia sclerotiorum*, *cinnamoyl-CoA reductase*, lignin, resistance

## Abstract

*Sclerotinia sclerotiorum* causes severe yield and economic losses for many crop and vegetable species, especially *Brassica napus*. To date, no immune *B. napus* germplasm has been identified, giving rise to a major challenge in the breeding of *Sclerotinia* resistance. In the present study, we found that, compared with a *Sclerotinia*-susceptible line (J902), a *Sclerotinia*-resistant line (J964) exhibited better xylem development and a higher lignin content in the stems, which may limit the invasion and spread of *S. sclerotiorum* during the early infection period. In addition, genes involved in lignin biosynthesis were induced under *S. sclerotiorum* infection in both lines, indicating that lignin was deposited proactively in infected tissues. We then overexpressed *BnaC.CCR2.b*, which encodes the first rate-limiting enzyme (cinnamoyl-CoA reductase) that catalyzes the reaction of lignin-specific pathways, and found that overexpression of *BnaC.CCR2.b* increased the lignin content in the stems of *B. napus* by 2.28–2.76% under normal growth conditions. We further evaluated the *Sclerotinia* resistance of *BnaC.CCR2.b* overexpression lines at the flower-termination stage and found that the disease lesions on the stems of plants in the T_2_ and T_3_ generations decreased by 12.2–33.7% and 32.5–37.3% compared to non-transgenic control plants, respectively, at 7days post-inoculation (dpi). The above results indicate that overexpression of *BnaC.CCR2.b* leads to an increase in lignin content in the stems, which subsequently leads to increased resistance to *S. sclerotiorum*. Our findings demonstrate that increasing the lignin content in the stems of *B. napus* is an important strategy for controlling *Sclerotinia*.

## Introduction

*Sclerotinia sclerotiorum* is a necrotrophic phytopathogenic fungus that obtains nutrients from plants by killing host cells and destroying host tissue, causing significant yield losses and economic damage to many crop and vegetable plants, especially *Brassica* crops (Seifbarghi et al., 2017; Li et al., 2018). Oilseed rape (*Brassica napus*) is one of the most important oil crop species, and its leaves, stems, and pods become severely rotted following infection with *S. sclerotiorum*, resulting in 10–20% yield losses every year in China. In addition to reducing yields, *S. sclerotiorum* also affects the oil and fatty acid contents of rapeseed, which leads to a decline in rapeseed products (Zhang et al., 2015a). Although improvements in cultivation measures and the application of chemical pesticides can somewhat reduce the impact of *S. sclerotiorum* disease, the effect is limited, and the use of chemical pesticides can also cause severe problems related to the “3Rs” (residue, resistance, and resurgence). Therefore, breeding new rapeseed varieties to combat disease resistance have become one of the most economical and effective methods.

Plants employ a series of defense mechanisms to protect themselves against pathogen attack through complex perception, transduction, and exchange of signals (Verhage et al., 2010). A commonly used model of the plant immune system is known as pathogen-associated molecular pattern (PAMP)-triggered immunity (PTI). In PTI, conserved molecules or structures of pathogens are perceived by plant pattern recognition receptors, followed by activation of defense responses. To circumvent PTI, pathogens deliver effector proteins inside host cells, which results in the initiation of a second level of defense called effector-triggered immunity (ETI; Jones and Dangl, 2006; Naveed et al., 2020). PTI and ETI responses overlap considerably, including oxidative bursts, transcriptional reprogramming, and the deposition of phenolic compounds, such as lignin (Nicholson and Hammerschmidt, 1992; Lee et al., 2019). Lignification of the plant cell wall has been suggested to be a physical barrier against pathogens (Nicholson and Hammerschmidt, 1992; Miedes et al., 2014; Liu et al., 2018; Lee et al., 2019). Lignin generally plays an important role in modifying the mechanical properties of cell walls by increasing cell wall rigidity to limit the diffusion of toxins from pathogens to hosts, nutrients from hosts to pathogens, and polysaccharide degradation by exogenous enzymes (Vorwerk et al., 2004; Eynck et al., 2012; Miedes et al., 2014). It is an amorphous phenolic heteropolymer resulting from the oxidative polymerization of at least two units of cinnamyl alcohol (monolignol) p-coumaryl, coniferyl and sinapyl alcohol, forming p-hydroxyphenyl (H), guaiacyl (G), and syringyl (S) lignin, respectively (Ros Barceló, 1997).

Previous studies have reported that increasing plant lignin could protect plants from the invasion and spread of pathogens. Overexpression of the wheat *TaRac1* (*Ras-related C3 botulinum toxin substrate 1*) gene in tobacco increases the lignin content and enhances resistance to tobacco black shank and bacterial wilt diseases (Ma et al., 2017). In cotton, overexpression of the *GhDIR1* (*dirigent1*) gene results in an increased lignin content, which blocks the spread of the fungal pathogen *Verticillium dahlia* (Shi et al., 2012). In *B. napus*, cell wall reinforcement by lignin was confirmed as a relevant factor for effective plant defense against attack of *S. sclerotiorum* (von Tiedemann et al., 2021). In the lignin synthesis process, phenylpropanoid metabolites are important secondary compounds, and some enzymes, including phenylalanine ammonia lyase (PAL), p-coumarate 3-hydroxylase (C3H), cinnamate-4-hydroxylase (C4H), hydroxycinnamoyltransferase (HCT), 4-coumarate:CoA ligase (4CL), caffeic acid O-methyltransferase (COMT), caffeoyl-CoA O-methyltransferase (CCoOMT), cinnamyl alcohol dehydrogenase (CAD), and cinnamoyl-CoA reductase (CCR; Whetten and Sederoff, 1995; Dixon et al., 2001; Wadenbäck et al., 2008; Li et al., 2014), have been shown to be involved in the synthesis of phenylpropanoids. What’s more, most of these enzyme-encoding genes have been reported to influence resistance to pathogens by participating in lignin synthesis. In *Camelina sativa*, the expression of the *CsCCR2* gene is strongly induced by *S. sclerotiorum*, which increases lignin synthesis and the resistance to *S. sclerotiorum* (Eynck et al., 2012). In maize, *ZmCCoAOMT2* might affect resistance to multiple pathogens by participating in lignin biosynthesis (Wang and Balint-Kurti, 2016; Yang et al., 2017). In *Arabidopsis*, the *CAD5* gene involved in lignin biosynthesis is an essential component of the defense against virulent and avirulent strains of the bacterial pathogen *Pseudomonas syringae* pv. *tomato* (Tronchet et al., 2010). Therefore, studying the expression and regulation of lignin-related genes to increase the lignin content in plants and enhance their resistance to pathogens have important prospective applications and value.

Cinnamoyl-CoA reductase (EC 1.2.1.44) is a key enzyme that regulates the lignin synthesis branch of phenylpropanoid metabolism. The lignin precursor is reduced to a lignin monomer and polymerized during the key process of lignin production. CCR catalyzes the reduction of five cinnamoyl-CoA esters and generates corresponding cinnamaldehyde compounds that participate in the biosynthesis of lignin (Gayoso et al., 2010). The synthesis of all three lignin monomers involves the participation of CCR. When CCR function is lost, the five cinnamoyl-CoA esters are synthesized into phenolic substances, such as flavonoids, anthocyanins, and plant antitoxins. Therefore, researchers have proposed that CCR is the first rate-limiting enzyme that catalyzes the reaction in lignin-specific pathways and has a potential regulatory effect on carbon flow through the lignin biosynthetic pathway (Menden et al., 2007). In general, downregulation of *CCR* leads to a decreased lignin content and changes in the lignin composition. An analysis of poplar *CCR2* knockout lines showed that the total lignin content decreased by approximately 10% compared to that of the wild type line (De Meester et al., 2020). Downregulation of *CCR1* expression in transgenic perennial ryegrass plants reduces the lignin composition of stems by reducing the levels of all three types (S, G, and H) of subunits (Tu et al., 2010). The acid-insoluble lignin (AIL) content in maize is significantly decreased in *ccr1* plants compared to wild-type plants (Smith et al., 2017). Based on previous results, researchers have confirmed that members of the *CCR* gene family are key enzyme-encoding genes controlling lignin synthesis. In addition, the expression of some *CCR* genes is induced by stress to enhance lignification, thus improving their resistance (Lauvergeat et al., 2001; Eynck et al., 2012; Srivastava et al., 2015; Li et al., 2016). Therefore, it is an effective way to change the plant lignin content by regulating the expression of *CCR* genes.

In this study, we found that a high lignin content in the stems of *B. napus* may limit the invasion and spread of *S. sclerotiorum*. We generated transgenic *B. napus* plants that overexpressed *BnaC.CCR2.b*, a key gene involved in the lignin monomer synthesis pathway. Overexpression of *BnaC.CCR2.b* led to an increase in the lignin content in the stems, which subsequently increased resistance to *S. sclerotiorum*. Based on our results, increasing the lignin content can improve the resistance of *B. napus* to *S. sclerotiorum*.

## Materials and Methods

### Construction of the Gene Phylogenetic Tree

The *CCR* genomic sequences in *B. napus* *Arabidopsis thaliana*, *Brassica rapa*, and *Brassica oleracea* were aligned using ClustalW with the default settings. Phylogenetic trees were constructed using the neighbor-joining method implemented in MEGA 7.0 software. The bootstrap test was executed with 1,000 replications (Larkin et al., 2007).

### Vector Construction and Genetic Transformation of *B. napus*

The *BnaC.CCR2.b* (BnaC06g40190D) gene was cloned from the cDNA library of *B. napus* cultivar J964, a *Sclerotinia*-resistant line, using BnCCR2-F/R primers (Supplementary Table S1). A fragment of *BnaC.CCR2.b* was then subcloned into PMDC83 using *Spe*I+*Asc*I double digestion, generating a *35S:BnaC.CCR2.b* vector. The *35S:BnaC.CCR2.b* vector construct was then introduced into *Agrobacterium tumefaciens* strain GV3101 for genetic transformation. The *B. napus* line J9712 (a susceptible line), which was kindly provided by Professor Yongming Zhou (National Key Laboratory of Crop Genetic Improvement, Huazhong Agricultural University), was used as a receptor. *B. napus* was transformed using the *A. tumefaciens*-mediated hypocotyl method. Selected plump seeds were surface sterilized with a 2% NaClO solution and subsequently rinsed with sterile distilled water. The seeds were germinated on half-strength Murashige and Skoog (MS) basal media supplemented with 2% sucrose in darkness. The seedlings were subsequently grown at 25°C in the dark for 7days. Afterward, the hypocotyl (~15mm) was cut, and the explants were floated in infection media [MS media supplemented with 3% sucrose and 100μm acetosyringone (AS); pH 5.8] for 20min. The explants were then transferred to cocultivation media (MS media supplemented with 3% sucrose, 1mg/l 2,4-dichlorophenoxyacetic acid (2,4-D), 0.3mg/l kinetin, 100μm AS, and 8g/l agar; pH 5.8) and incubated for 3days. The explants were subsequently transferred to MS callus induction media supplemented with 3% sucrose, 1mg/l 2,4-D, 0.3mg/l kinetin, 5mg/l AgNO_3_, 300mg/l timentin, 25mg/l hygromycin B (Hyg), and 8g/l agar (pH 5.8) and incubated at 25°C for 20days. The explants were then transferred to shoot differentiation media [MS media supplemented with 1% glucose, 100μm AgNO3, 2.0mg/l zeatin, 0.1mg/l indoleacetic acid (IAA), 300mg/l timentin, 25mg/l Hyg, and 8g/l agar; pH 5.8] and incubated until shoot growth was initiated (fresh MS media was replaced every 20days). Healthy green shoots were ultimately transferred to bottles containing root induction media (MS media supplemented with 1% sucrose and 8g/l agar; pH 5.8), after which the plantlets acclimated and became established. Total DNA was extracted from the young leaves of each transgenic plant using the cetyltrimethylammonium bromide (CTAB) method, after which PCR was performed to identify positive transformants using the specific primers 35S-3 and BnCCR2-R (Supplementary Table S1).

### Histological Staining of Lignin

Histological staining of lignin was performed using Wiesner reagent (Li et al., 2013). Wiesner stain is known to react with cinnamaldehyde residues in lignin, and the color intensity is consistent with the total lignin content. Whole cross-sections and manually cut cross-sections of stems were obtained at the same position (approximately 30–40cm above the ground) at the initial, full, and final flowering stages. The stem sections were treated with a 1% phloroglucinol alcohol solution for 2min, followed by the application of a drop of concentrated hydrochloric acid (32%). The manually cut cross-sections were viewed and imaged (bright field) using a Zeiss AxioPlan 2 microscope.

### Determination of the Lignin Content in Stems

Stem tissues of *B. napus* lines J964 and J902 were collected at the final flowering stage. Stem tissues of the transgenic lines in the T_2_ generation were collected at the mature stage, because of the requirement for seed harvesting. Stems at a length of approximately 30cm of six individuals of each accession in each replicate were cut at 20cm above the ground using a sharp knife. The samples were then dried at 60°C, cut into small pieces, ground into powder with a grinder, filtered through an 80-mesh screen (0.15×0.15mm), and then stored in a dry container. The total lignin content was determined by a two-step acid hydrolysis method, with modifications (Xu et al., 2012). The lignin consisted of both acid-insoluble lignin (AIL) and acid-soluble lignin (ASL).

ASL: A 0.3g sample was recorded as W_1_. The sample was extracted with benzene-ethanol (67/33, v/v) in a Soxhlet incubator for 4h and then air dried in a fume hood. The sample was hydrolyzed with 10ml of 67% H_2_SO_4_ (v/v) in a shaker at 30°C for 1.5h. Afterward, 200ml of ddH_2_O were added for hydrolysis, which was performed at 120°C for 1h. After hydrolysis, the hydrolysis liquor was transferred to a 250ml volumetric flask and brought up to 250ml (V) with 2.88% sulfuric acid. The absorbance was between 0.2 and 0.7, and the dilution factor was denoted as D. The absorbance of the sample was read at 205nm *via* UV–Vis spectroscopy, and 2.88% sulfuric acid was used as a blank. The amount of ASL was calculated as follows: ASL (%)=A×D×V/(1,000×K×W_1_)×100, where A is the absorption value, D is the dilution ratio of the sample, V is the total volume of the filtrate, and K is the absorptivity constant (110l/g/cm).

ASL: The hydrolysis liquor obtained previously was filtered through a G3 crucible filter, and the total residue was then transferred to a crucible filter. The acid-insoluble residue was dried in an oven at 60°C until a constant weight was achieved. The samples were then removed from the oven and cooled in a dry container. The weight of both the crucible and the dry residue was recorded (W_2_). The dried residue was ultimately ashed in a muffle furnace at 200°C for 30min followed by 575°C for 4h. The crucibles and ash were subsequently weighed, and the weight (W_3_) was recorded. The AIL of the original sample was calculated as follows: AIL (%)=(W_2_−W_3_)×100/W1. The total lignin (%)=ASL%+AIL%. The lignin content was determined from 300mg of extract-free dry stems for three biological replicates. The lignin content was calculated as the weight percent of the dry extract-free stems. All experiments were conducted with three replications.

### Assessment of Resistance to *S. sclerotiorum*

*Sclerotinia sclerotiorum* isolate SS-1 was maintained and cultured on potato dextrose agar (PDA) media (Wu et al., 2019). Under natural field conditions, stems were inoculated with *S. sclerotiorum* to evaluate its resistance at the termination of flowering. Approximately 10 stems of each transgenic line (T_2_ and T_3_) in each replicate were inoculated at a height of 40–50cm above the ground with mycelial agar plugs (5mm diameter). Each plug was affixed with plastic wrap to ensure close contact of the inoculum with the stem surface and to maintain a high humidity. The lesion length along the stems was measured at 7days post-inoculation (dpi). This assessment was conducted for three replications.

### Quantitative Real-Time PCR Analyses of Extracted RNA

To examine the expression level of *BnaC.CCR2.b* in the transgenic lines, the stem tissues of plants in the T_0_ and T_1_ generations were collected and then quickly put into liquid nitrogen for RNA extraction. The stem tissues of the plants in the T_1_ generation were collected (three biological replicates), and each replicate consisted of three stem tissues from three different plants. qBnaC.CCR2.b-F/R primers (Supplementary Table S1) were used to measure the expression of *BnaC.CCR2.b.*

To detect whether overexpression of *BnaC.CCR2.b* in *B. napus* would alter the expression levels of genes related to lignin synthesis, J9712 plants and overexpression plants (T_2_ generation) were selected for inoculation and sampling (three biological replicates). When the plants were at the flower termination stage, three sites on the primary stem were inoculated at three consecutive internodes (approximately 30–60cm above the ground) with 5mm diameter mycelial agar plugs. Epidermal stem tissues extending 10mm beyond the inoculation site and 1mm deep were sampled. Each replicate involved 12 plants per line at three different time points (0, 48, and 72 hpi).

Total RNA was extracted using a Plant Total RNA Extraction Kit (BioTeke, China) following the manufacturer’s instructions and then treated with RNase-free DNase I (Thermo Scientific, United States) to remove genomic DNA contaminants. The RNA was transcribed into cDNA using the HiScript^®^ QRT Super Mix for qPCR (Vazyme, China). The expression levels of the key lignin synthesis pathway genes *BnPAL2*, *BnPAL1*, *BnC4H*, *Bn4CL1*, *BnHC*, *BnC3H*, *BnCCoAOM*, *BnFAH1*, *BnCCR1*, *BnCCR2*, *BnCOMT*, *BnCAD4*, and *BnCAD5* were measured *via* qRT-PCR. The primers were designed such that the sequences of all copies of each gene were incorporated (Supplementary Table S1). qRT-PCR was performed for three technical replicates. The *BnUBC9* gene was chosen as the reference gene. Normalization was then performed, and the relative expression was calculated to analyze the qRT-PCR data.

### Transcriptome Sequencing of Various Tissue Samples of *B. napus* Line J9712

Expression patterns of *BnCCR* genes in various tissues of *B. napus* line J9712 were determined by transcriptome sequencing. Tissue samples were collected at different growth stages. Cotyledon, seedling root, stem, rosette leaf, cauline leaf, shoot apical meristem, flower bud, unpollinated ovary, seed, and silique walls at different stages (14days, 24days, 34days, and 50days after flowering) were collected from five plants in each biological replicate. Three biological replicates were performed. All samples were sequenced using an Illumina HiSeq X Ten sequencer. The sequencing was performed as paired-end reads that were 2× 150bp in length. The original data set was deposited in the NCBI Sequence Read Archive (accession no. PRJNA749379). RNA sequence analysis was performed as described in our previous study (Wu et al., 2016).

## Results

### A High Content of Lignin in the Stems of *B. napus* May Limit the Invasion and Expansion of *S. sclerotiorum*

In our previous study, we performed dynamic transcriptomic analyses to understand the different defense responses to *S. sclerotiorum* in a resistant *B. napus* line (J964) and a susceptible *B. napus* line (J902) at 24, 48, and 96h post-inoculation (hpi). We found that differences between J964 and J902 were associated with differences in the magnitude of gene expression changes, which were detected mainly at 48 or 96 hpi. At 24 hpi, only 122 genes were slightly upregulated in J964, while 4,129 genes were upregulated in J902 (Wu et al., 2016). GO enrichment analysis showed that half of the top 30 enriched GO terms in the biological process for the 4,129 upregulated genes in J902 belonged to the secondary GO category, response to stimulus (Supplementary Figure S1), suggesting that the defense response was faster in J902 than in J964. However, *S. sclerotiorum* infected and propagated more easily in J902 than in J964. This finding suggests that in addition to the second line of defense (the plant immune system), the first line of defense (the passive defense line) may also differ between the two lines.

**Figure 1:**
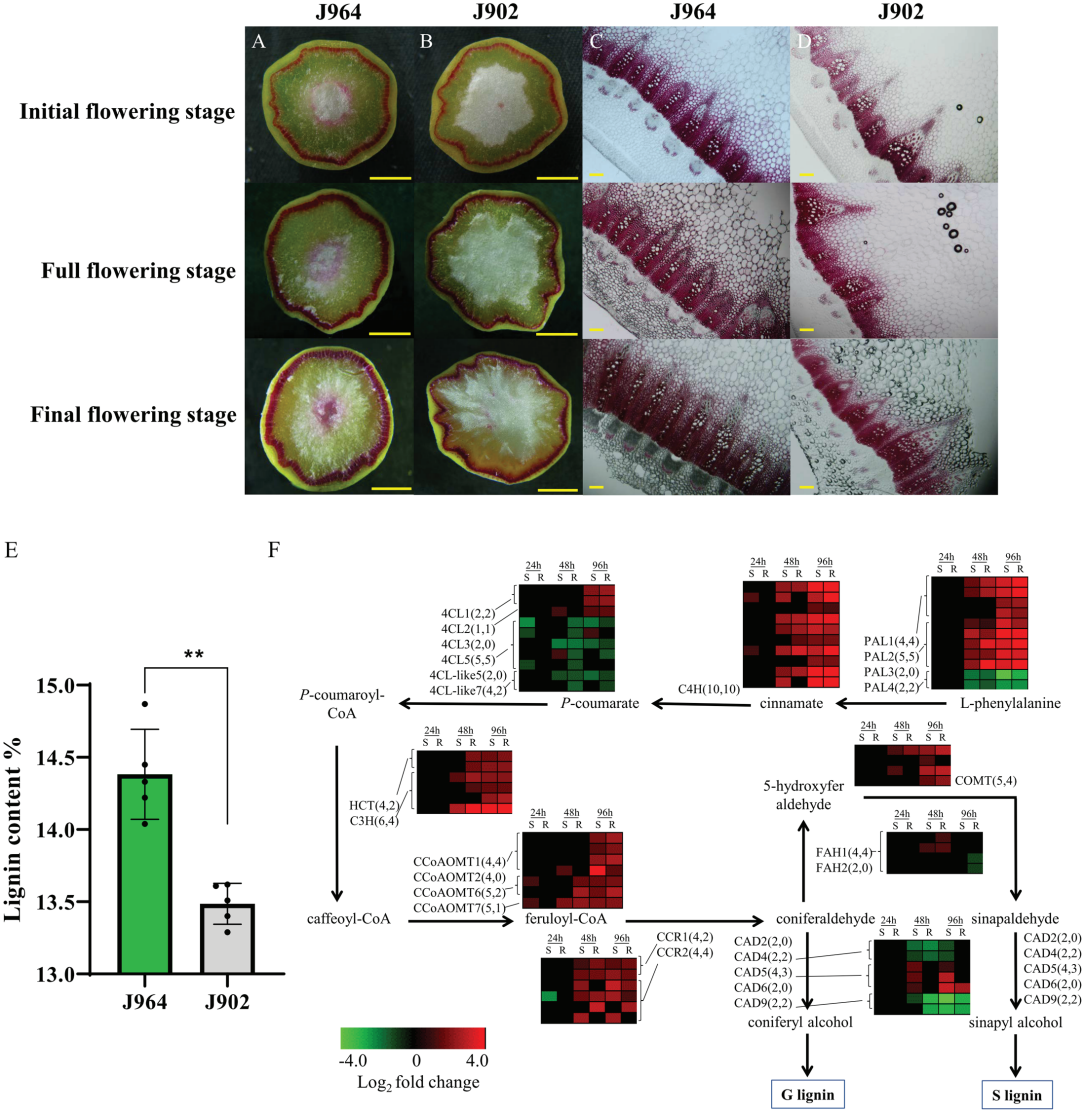
Lignin content and expression of genes related to lignin synthesis in the stems of a *Sclerotinia*-resistant line (J964) and a *Sclerotinia*-susceptible line (J902). **(A–D)** Phloroglucinol-HCl staining of lignin in cross-sections of J964 and J902 stems at different flowering stages. The scale bars represent 1cm in the whole cross-sections in A-B and 500μm in the manually obtained cross-sections in C,D. **(E)** Percentage of lignin content in the stems of J964 and J902. The values are presented as the means ± SDs of five biologically independent replicates. The asterisks represent statistically significant differences (^**^p<0.01; Student’s t-test). **(F)** Whole-genome-wide comparison of genes involved in monolignol biosynthesis pathways in J964 and J902 after *S. sclerotiorum* infection. The copy numbers of the genes and the differentially expressed genes are listed in square brackets.

To determine whether the stem lignin differed before inoculation, the stem cross-sections of the two lines were examined carefully. Interestingly, a significant difference was found after the stems were stained with Wiesner reagent to detect lignin. Both lines presented deep magenta staining in the xylem, but J964 presented better xylem development and deeper lignin staining than J902 (Figures 1A,B). In J964, the vascular bundles were tightly arranged and had developed neatly, and the water content of the pith was high (Figure 1C). However, in J902, the vascular bundles were loosely arranged and unevenly developed. Even the xylem of some vascular bundles was degraded, and the pith lost a large amount of water and began to soften (Figure 1D). As expected, the stem lignin content in J964 was significantly higher than that in J902 (Figure 1E). Hence, the better development of xylem and the higher content of lignin in the stems seem to limit the invasion and spread of *S. sclerotiorum*.

In addition, according to the transcriptome data, genes involved in lignin biosynthesis were induced after *S. sclerotiorum* infection (Figure 1F). However, no obvious difference in gene expression patterns between the two lines was observed (Figure 1F). These results indicate that lignin synthesis pathway was induced in both lines under the infection of *S. sclerotiorum* and lignin was deposited proactively in infected tissues, possibly in an attempt to limit pathogen colonization. Therefore, we overexpressed genes involved in lignin biosynthesis in *B. napus* to determine whether the lignin content in the stems could be increased to improve resistance to *S. sclerotiorum*. Then, we chose the *CCR* gene for overexpression in *B. napus*. This gene encodes the first rate-limiting enzyme that catalyzes the reaction of lignin-specific pathways (Menden et al., 2007).

### Phylogenetic and Expression Pattern Analyses of the *BnCCR1* and *BnCCR2* Genes

**Figure 2:**
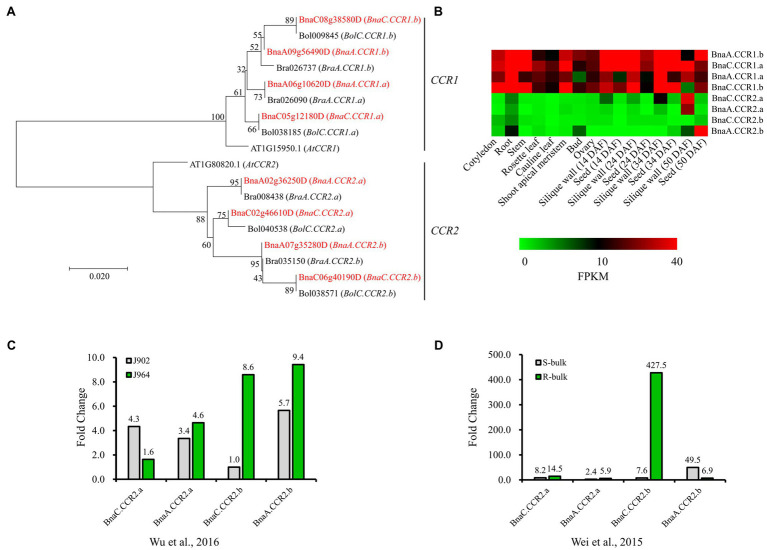
Phylogenetic and expression analyses of *BnCCR* genes. **(A)** Phylogenetic relationships of *CCR* genes from *Brassica napus*, *Brassica rapa*, *Brassica oleracea*, and *A. thaliana.* The red color indicates genes from *B. napus*. Bootstrap values (1,000 replications) are shown at each branch as percentages. A branch length scale bar is shown beneath the tree. **(B)** Expression patterns of *BnCCR* genes in various tissues of *B. napus* line J9712, as determined by transcriptome sequencing. FPKM, fragments per kilobase of transcript per million fragments mapped. DAF, days after flowering. **(C,D)** Expression patterns of *BnCCR* genes in the stems of *B. napus* at 48h after *Sclerotinia sclerotiorum* infection. The fold changes (inoculated/mock inoculated) were calculated through transcriptomic analyses performed by Wu et al. (2016) and Wei et al. (2015). J964: a resistant line. J902: a susceptible line. R- and S-bulk: mixed pools of resistant and susceptible lines, respectively.

To better understand the evolutionary history and functions of *CCR* in Brassicaceae, we searched for all possible copies of *CCR* in the released reference genome sequence of Darmor-bzh *B. napus*, with *Arabidopsis AtCCR1* (AT1G15950.1) and *AtCCR2* (AT1G80820.1) gene sequences used as queries. For both *AtCCR1* and *AtCCR2*, four close homologs were identified in *B. napus* (Figure 2A). We named these *AtCCR1* homologs *BnaA.CCR1.a* (BnaA06g10620D), *BnaA.CCR1.b* (BnaA09g56490D), *BnaC.CCR1.a* (BnaC05g12180D), and *BnaC.CCR1.b* (BnaC08g38580D), which were 98.2, 97.1, 98.0, and 96.5% similar, respectively, to *AtCCR1* at the amino acid level. Similarly, four *AtCCR2* homologs were named *BnaA.CCR2.a* (BnaA02g36250D), *BnaA.CCR2.b* (BnaA07g35280D), *BnaC.CCR2.a* (BnaC02g46610D), and *BnaC.CCR2.b* (BnaC06g40190D), whose sequences were 97.9, 96.4, 97.6, and 95.8% homologous, respectively, to that of *AtCCR2*. Homology analysis indicated that the *BnCCR1* and *BnCCR2* genes may have conserved functions similar to those of *AtCCR1* and *AtCCR2*, respectively. In addition, for each *BnCCR* gene, the closest homologous gene was found in the corresponding progenitor genomes (*B. oleracea* and *B. rapa*; Figure 2A), suggesting that *BnCCR* genes may not have undergone duplication or deletion after *B. napus* formation due to recent allopolyploidy between ancestors of *B. oleracea* and *B. rapa*.

The transcriptome data indicated that *BnCCR1* genes were highly expressed in all tissues of *B. napus*, while the *BnCCR2* genes were not expressed or expressed at low levels in most tissues (Figure 2B). However, all four copies of *BnCCR2* were induced after *S. sclerotiorum* infection, while only two copies of *BnCCR1* were moderately induced (Figure 1F). These results are consistent with those of previous studies in *Arabidopsis* showing that *AtCCR1* and *AtCCR2* are differentially expressed during development and in response to pathogen infection (Lauvergeat et al., 2001). Most *BnCCR2* genes were expressed to a much greater degree in the resistant line J964 or resistant bulk (mixed pools of resistant lines) group than in the susceptible line J902 or susceptible bulk (mixed pools of susceptible lines) group at 48 hpi (Figures 2C,D). The most strongly induced *BnCCR2* gene was *BnaC.CCR2.b*, whose expression increased 427.5-fold in the resistant bulk group but only 7.6-fold in the susceptible bulk group (Figure 2D). These findings suggest that *BnCCR2* genes, especially *BnaC.CCR2.b*, are important for *Sclerotinia* resistance.

### Generation of *BnaC.CCR2.b*-Overexpressing *B. napus* Lines

**Figure 3:**
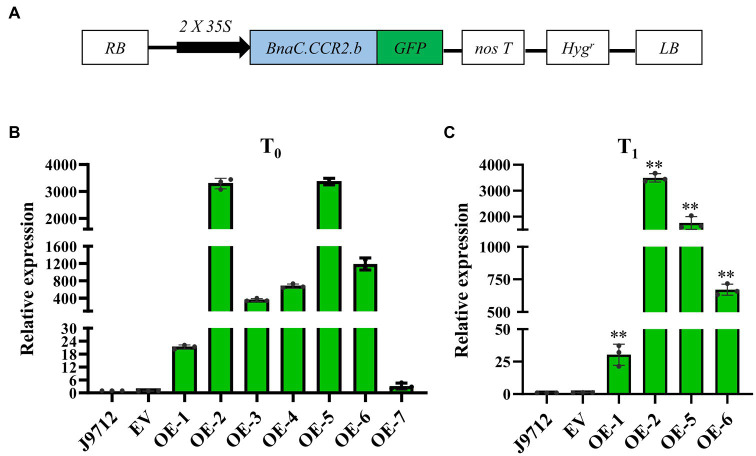
Schematic of the *BnaC.CCR2.b* overexpression construct and expression analysis of *BnaC.CCR2.b* in transgenic plants. **(A)** Schematic diagram of the *BnaC.CCR2.b* overexpression construct. *35S*: cauliflower mosaic virus 35S promoter. *GFP*: green fluorescent protein*. NosT*: nopaline synthase gene terminator. *Hyg^r^*: *hygromycin B* resistance gene. RB and LB represent the right and left borders, respectively. **(B)** and **(C)** Expression of *BnaC.CCR2.b* in the stems of T_0_ and T_1_ transgenic lines, respectively. J9712, transgenic receptor line. EV, pMDC83 empty vector transgenic line. OE-1 to OE-7 are *BnaC.CCR2.b* overexpression lines. The asterisks indicate significant differences between the control and transgenic lines (^*^p<0.05; ^**^p<0.01; and Student’s t-test).

To investigate whether overexpression of *BnaC.CCR2.b* in *B. napus* can increase resistance to *S. sclerotiorum*, we introduced the *BnaC.CCR2.b* gene into *B. napus* line J9712 (a susceptible line) *via* a pMDC83 binary expression vector containing a *hygromycin B* resistance gene and the *BnaC.CCR2.b* gene under the control of the CaMV 35S promoter (Figure 3A). The *35S:BnaC.CCR2.b* vector was subsequently transformed into *B. napus via Agrobacterium*-mediated transformation. Seven independent overexpression transgenic lines of the T_0_ generation were ultimately obtained (named OE-1 to OE-7). We performed qRT-PCR to measure the expression of *BnaC.CCR2.b* in the transgenic lines and found that its expression in all seven transgenic lines was higher than that in J9712 and empty vector (EV)-transformed control plants (Figure 3B). The expression level of *BnaC.CCR2.b* in the OE-1/2/5/6 transgenic lines of the T_1_ generation was further measured, the results of which were consistent with the results in the T_0_ generation (Figure 3C). The OE-1/2/5/6 transgenic lines with high and stable expression of *BnaC.CCR2.b* were selected for subsequent experiments.

### Overexpression of *BnaC.CCR2.b* Increases Resistance to *S. sclerotiorum*

**Figure 4:**
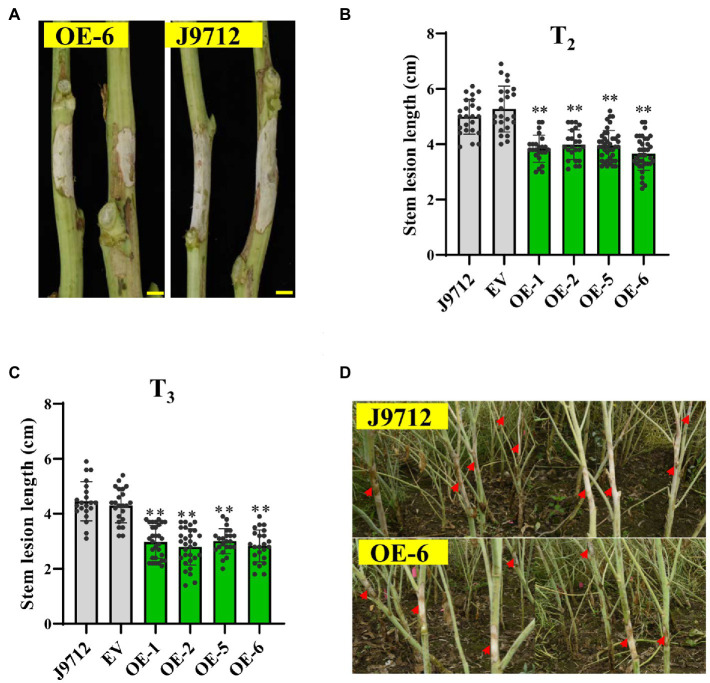
Assessment of the disease resistance of *BnaC.CCR2.b* overexpression lines against *S. sclerotiorum* by stem inoculation in the field. **(A)** Representative images of disease lesions on the stems of OE-6 lines in T_2_ generation at 7 dpidays post-inoculation Bar=1cm. **(B,C)** Stem lesion length in T_2_ (B)- and T_3_ (C)-generation transgenic plants at 7 dpi. The values are presented as the means ± SDs (*n*=25–30 in T_2_ and T_3_). The asterisks indicate significant differences between the overexpression transgenic lines and the J9712 lines (^*^p<0.05; ^**^p<0.01; and Student’s t-test). **(D)** Representative images of disease lesions on the stems at 20 days post-inoculation.

To further confirm whether *BnaC.CCR2.b* is related to resistance to *S. sclerotiorum*, we evaluated the *Sclerotinia* resistance of *BnaC.CCR2.b* overexpression lines in the T_2_ and T_3_ generations at the flower-termination stage. The stem inoculation experiment results showed that, compared with those of J9712 and the EV-transformed plants, the stem lesions of the plants of the overexpression lines expanded more slowly (Figure 4A). Statistical analysis showed that, compared with that of the J9712 line, the stem lesion length of the OE-1/2/5/6 transgenic lines in the T_2_ and T_3_ generations was reduced by 12.2–33.7% and 32.5–37.3%, respectively, at 7 dpi (Figures 4B,C). The lesion expanded further to a large part of the stem of J9712 at 20 dpi, while the lesion was restricted to approximately 6–8cm on the stems of the overexpression plants (Figure 4D). Taken together, the above results indicate that *BnaC.CCR2.b* positively regulates *B. napus* resistance to *S. sclerotiorum*.

### Overexpression of *Bnac.CCR2.b* Increases Lignin Accumulation in the Stems of *B. napus*

**Figure 5:**
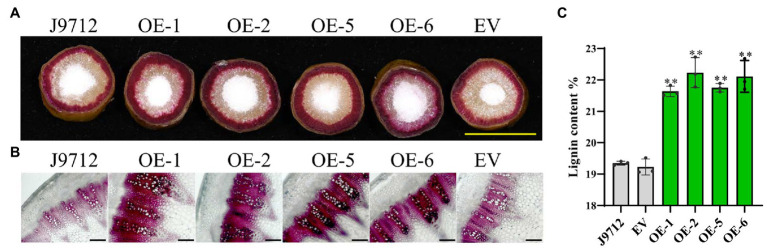
Detection of lignin accumulation in the *BnaC.CCR2.b* overexpression lines. **(A)** Phloroglucinol-HCl staining of lignin in whole cross-sections. The scale bars represent 1cm. **(B)** Phloroglucinol-HCl staining of lignin in manually obtained cross-sections. The scale bars represent 100μm. J9712, transgenic receptor line. EV, empty vector transgenic line. OE-1/2/5/6, *BnaC.CCR2.b* overexpression lines (T_2_ generation). **(C)** Percentage of lignin content in stems of *BnaC.CCR2.b* overexpression lines, J9712 lines, and EV lines. The values are presented as the means ± SDs of three biologically independent replicates. The asterisks indicate significant differences between the overexpression transgenic lines and the J9712 lines (^*^p<0.05; ^**^p<0.01; and Student’s t-test).

To determine whether overexpression of *BnaC.CCR2.b* in *B. napus* would change the accumulation of lignin, cross-sections of the stems of overexpression lines (T_2_) and control plants at the flower-termination stage were strained with Wiesner reagent to evaluate the lignin deposition. Phloroglucinol-HCl staining of lignin in whole cross-sections showed that the staining was more intense in the overexpression transgenic lines than in the J9712 plants and EV-transformed lines (Figure 5A). Staining of the manually cut cross-sections revealed that, in the overexpression transgenic lines, the number of vascular bundles did not change significantly; however, individual vascular bundles became wider, the xylem layer became thicker, and the staining was more intense in the overexpression lines compared with the J9712 and EV lines (Figure 5B). Chemical analysis indicated that the overexpression transgenic lines had a higher lignin content (21.6–22.2%) than the J9712 plants (19.3%) and EV-transformed lines (19.2%; Figure 5C). Taken together, these results indicate that the overexpression of *BnaC.CCR2.b* can significantly increase the lignin accumulation in the stems of *B. napus*, which might thus limit the invasion and spread of *S. sclerotiorum*.

In addition, the agronomic traits of *BnaC.CCR2.b*-overexpressing plants in the T_2_ generation were investigated, and no significant differences between the transgenic plants and J9712 plants were detected (Supplementary Table S2).

### Expression Analysis of Genes Related to Lignin Synthesis

**Figure 6:**
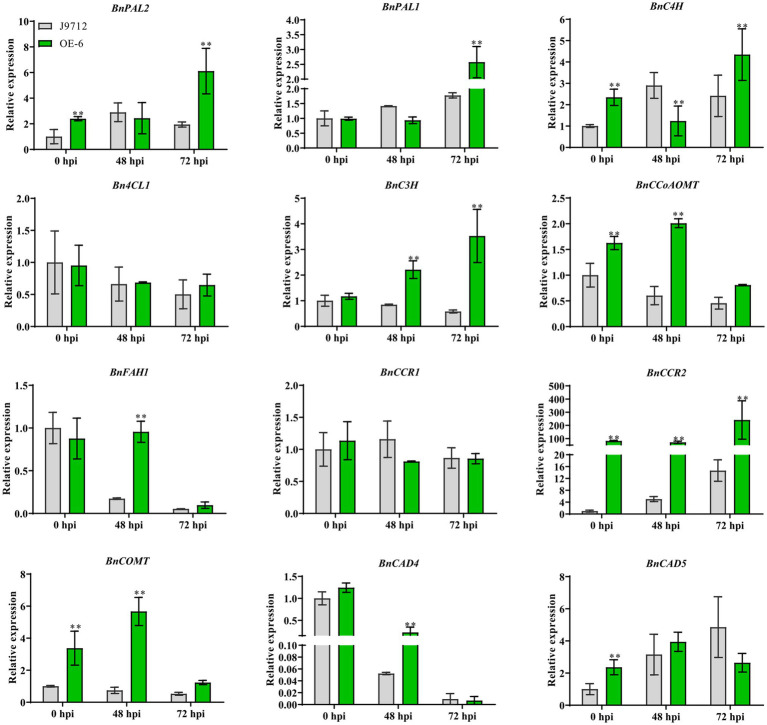
Expression analysis of key genes involved in lignin biosynthesis under normal growth conditions (before inoculation) and *S. sclerotiorum* inoculation conditions (48 hpi and 72 hpi). The bars represent the standard deviations (*n*=3). ^*^ and ^**^ indicate significant differences between OE-6 and J9712 (^*^p<0.05; ^*^p<0.01; and Student’s t-test).

To assess whether overexpression of *BnaC.CCR2.b* in *B. napus* alters the expression of genes in the lignin synthesis pathway, the expression levels of key lignin synthesis pathway genes *BnPAL2*, *BnPAL1*, *BnC4H*, *Bn4CL1*, *BnC3H*, *BnCCoAOM*, *BnFAH1*, *BnCCR1*, *BnCCR2*, *BnCOMT*, *BnCAD4*, and *BnCAD5* were measured *via* qRT-PCR analyses. The results showed that the expression of most of the lignin synthesis-related genes (*BnPAL2*, *BnC4H*, *BnC3H*, *BnCCoAOM*, *BnCCR1*, *BnCOMT*, and *BnCAD5*) was upregulated in the stems of OE-6 plants compared with J9712 plants under normal growth conditions (before inoculation, 0 hpi; Figure 6). These results suggested that overexpression of *BnaC.CCR2.b* in *B. napus* induced the lignin synthesis pathway, which altered the lignin content in the stems of plants under normal growth conditions.

We further examined the expression of genes related to the lignin synthesis pathway in plants under infection with *S. sclerotiorum*. The results showed that the expression levels of genes related to lignin synthesis in the OE-6 and J9712 lines changed after *S. sclerotiorum* infection. In both lines, the differences in the levels of these genes were most pronounced from 48 to 72 hpi, and the number of differentially expressed genes was highest at 72 hpi. However, the expression levels of the *Bn4CL1* and *BnCCR1* genes appeared to be moderately altered. Overall, *BnCAD4* and *BnFAH1* were drastically downregulated in both lines from 48 to 72 hpi, while *BnPAL2*, *BnPAL1*, and *BnC4H* were upregulated (Figure 6). It was also found that the expression changes of some lignin biosynthesis-related genes (*BnPAL1*, *BnPAL2*, *BnC3H*, *BnCCoAOM*, *BnFAH1*, *BnCCR2*, and *BnCOMT*) were more significant in OE-6 than in J9712 (Figure 6), suggesting that the lignin synthesis pathway in the *BnaC.CCR2.b* overexpression line was induced more drastically. Taken together, these results suggest that *BnCCR2* is involved in pathogen-induced lignification, which is also important for increasing resistance to *S. sclerotiorum*.

## Discussion

Breeding of *Sclerotinia*-resistant *B. napus* poses a great challenge due to the limited availability of immune or highly resistant germplasms. *Sclerotinia* resistance is a quantitative characteristic controlled by large numbers of genes, although a lot of QTLs have been mapped, there are few major QTLs with large individual effects on *Sclerotinia* resistance (Wu et al., 2013, 2019). Additionally, although some resistance-related genes have been revealed by reverse genetics, the resistance mechanism is still confused (Ding et al., 2021). Despite worldwide efforts to breed *Sclerotinia*-resistant *B. napus*, the most effective strategy for controlling Sclerotinia rot currently relies on fungicide application, which is environmentally hazardous and costly. Hence, it is imperative to develop new feasible strategies to protect oilseed rape from *S. sclerotiorum*. Lignin participates in the thickening of the secondary cell wall, increases the stiffness and mechanical resistance of stems, prevents water penetration into cell walls, and protects plants from pathogen infection (Kesarwani et al., 2000; Tronchet et al., 2010; Eynck et al., 2012). Many studies have confirmed that lignin has a significant effect on the resistance to pathogens (Eynck et al., 2012; Shi et al., 2012; Ma et al., 2017; von Tiedemann et al., 2021). In the present study, we found that the high content of lignin in the stems of *B. napus* could increase resistance to *S. sclerotiorum*.

The key role of the *CCR* gene in lignin synthesis has been confirmed in previous studies. Changing the expression level of the *CCR* gene might significantly affect the lignin content in plants (Lüderitz and Grisebach, 1981; Escamilla-Treviño et al., 2010; Zhou et al., 2010; Hu et al., 2011; Ma et al., 2011; De Meester et al., 2020). These results indicated that controlling lignin content by regulating the expression of the key gene involved in the lignin synthesis pathway is an effective way to improve plant disease resistance. Our study showed that overexpression of *BnaC.CCR2.b* in *B. napus* significantly increased lignin accumulation in the stems and limited the expansion of *S. sclerotiorum* (Figures 4, 5). Our results further confirmed that the *BnCCR* gene plays an important role in lignin synthesis.

Two or more *CCR* homologous genes are present in most plant genomes. In *Arabidopsis*, two genes coding *CCR*s (*AtCCR1* and *AtCCR2*) are known to be differentially expressed. *AtCCR1* is involved in constitutive lignification, whereas *AtCCR2* is involved in pathogen-induced lignification (Lauvergeat et al., 2001; Fraser and Chapple, 2011). Studies on the Chinese white pear *PbCCR1* and *PbCCR2* genes have shown that *PbCCR1* and *PbCCR2* are somewhat functionally redundant, and both demonstrate the ability to participate in lignin biosynthesis. *PbCCR1* may be the major gene for lignin biosynthesis, while *PbCCR2* has little effect on lignin biosynthesis (Su et al., 2019). In switchgrass, for example, *PvCCR1* is involved mainly in lignin synthesis, and *PvCCR2* may function in defense (Escamilla-Treviño et al., 2010). In *Arabidopsis*, sorghum and maize, *CCR2* expression significantly increases after pathogen invasion, suggesting that the *CCR2* gene might be involved in the plant disease resistance response (Fraser and Chapple, 2011; Wang et al., 2015; Li et al., 2016). In our study, two homologous *CCR* genes, *BnCCR1* and *BnCCR2*, were found in *B. napus*. By analyzing the organization of the expression patterns of these two genes, we found that *BnCCR1* was expressed in all the various organs of rape (Figure 2B), speculating that *BnCCR1* was involved in constitutive lignification during the growth and development of *B. napus*, while all four copies of *BnCCR2* were strongly induced under *S*. *sclerotiorum* infection (Figures 2C,D), suggesting that *BnCCR2* genes might be important for *Sclerotinia* resistance. Therefore, we overexpressed the *BnaC.CCR2.b* gene in *B. napus* to determine whether *BnCCR2* is associated with resistance to *S. sclerotiorum*.

The increase or decrease in *CCR* gene expression can significantly affect the content and composition of lignin and ultimately affect various aspects of plant growth and development, such as plant height, seed quantity, stem thickness, and leaf morphology (Zhang et al., 2015b). For example, compared with their wild-type counterparts, *Arabidopsis ccr1* mutants exhibit a severe dwarf phenotype, and their rosettes are smaller. This phenotype can be restored for *ccr1 ProSNBE:CCR1* plants (De Meester et al., 2018). When CCR activity is reduced in tobacco, significant changes in plant height, leaf development, length of flowering period, seed quality, and other aspects occur (Prashant et al., 2011). In this study, we statistically analyzed the agronomic traits of transgenic lines and found that overexpression of the *BnaC.CCR2.b* gene did not affect the growth or development of *B. napus* (Supplementary Table S2). At the same time, the quality of seeds of the transgenic lines was evaluated, and the results showed that overexpression of the *BnaC.CCR2.b* gene had no significant effect on seed oil content or other qualities (Supplementary Table S2). An important practical consideration associated with altering specific lignin biosynthesis-related genes is obtaining modified-lignin properties without compromising growth and development. Our results showed that overexpression of the *BnaC.CCR2.b* gene in rapeseed altered the lignin content in the stems but had no effect on the growth and development of *B. napus* or the quality of its seeds.

In this study, we overexpressed the *BnCCR2* gene in *B. napus*, which effectively increased the lignin content in the stems and significantly enhanced resistance to *Sclerotinia*. However, research on the role of *CCR* genes in disease resistance with mutants or RNAi lines is still lacking. Many factors, such as physiological, biochemical, and molecular biological factors, must be further explored to fully characterize the role of the CCR gene in crop improvement.

## Conclusion

We found that the lignin content in the stems of *B. napus* is critical for resistance to *S. sclerotiorum*. Overexpression of *BnaC.CCR2.b* in *B. napus* significantly increased lignin accumulation in the stems and limited the spread of *S. sclerotiorum*, indicating an important role of *BnCCR2* in lignin improvement and disease resistance. Our findings revealed that increasing the content of lignin can improve *B. napus* resistance to *S. sclerotiorum*, which should facilitate the development of effective strategies for *Sclerotinia* resistance breeding.

## Data Availability Statement

The original contributions presented in the study are included in the article/Supplementary Material, further inquiries can be directed to the corresponding authors.

## Author Contributions

JW and DL designed the experiments and wrote the manuscript. QS, JL, and YW revised the manuscript. YW and JW supervised this project. LL, PL, SL, JL, and YW performed the experiments and analyzed the data. All authors read the manuscript and approved the content.

## Funding

This work was supported by the National Natural Science Foundation of China (32072020, 31901504, and 32172019), the Jiangsu Agricultural Science and Technology Innovation Fund (CX(20)3120), the National Science Foundation of Jiangsu Province (BE2018356 and BK20190894), the Project of Special Funding for Crop Science Discipline Development (yzuxk202006), the Priority Academic Program Development of Jiangsu Higher Education Institutions, and the Qinglan Project of Yangzhou University.

## Conflict of Interest

The authors declare that the research was conducted in the absence of any commercial or financial relationships that could be construed as a potential conflict of interest.

## Publisher’s Note

All claims expressed in this article are solely those of the authors and do not necessarily represent those of their affiliated organizations, or those of the publisher, the editors and the reviewers. Any product that may be evaluated in this article, or claim that may be made by its manufacturer, is not guaranteed or endorsed by the publisher.
