# Supplementary material for: Overexpression of Cinnamoyl-CoA Reductase 2 in Brassica napus Increases Resistance to Sclerotinia sclerotiorum by Affecting Lignin Biosynthesis
Source: Front Plant Sci. 2021 Sep 23;12:732733. doi: 10.3389/fpls.2021.732733 (PMC8494948; doi:10.3389/fpls.2021.732733)
Supplement: Supplementary file 1 [file Table_1.DOCX]

###### Supplementary information

**Table S1 List of primers used for qRT-PCR, gene cloning and positive transformants identification.**

| **Primer name** | **Sequence (5'-3')** | **Usage** |
| --- | --- | --- |
| qBn4CL1-F | AGCCTCGAACTCGAAGCTCATC | qRT-PCR for *Bn4CL1* |
| qBn4CL1-R | ACGTCGTCGGAAGAAATCTCC |  |
| qBnC3H-F | GTACTTAGGAGCGGTTGCG | qRT-PCR for *BnC3H* |
| qBnC3H-R | ATGTGTTCAGCTATCGACAGT |  |
| qBnC4H-F | GAGCTAGTGAACCATCCTGA | qRT-PCR for *BnC4H* |
| qBnC4H-R | AATCCCAGGACAGCTTCTACG |  |
| qBnCAD4-F | CTCAAGGTGGCTTCGCTGACA | qRT-PCR for *BnCAD4* |
| qBnCAD4-R | TCTTCACACCCATGTGTCCAA |  |
| qBnCAD5-F | GAACAGTATTGTCCCAAGAA | qRT-PCR for *BnCAD5* |
| qBnCAD5-R | AGCTTATGACAGTCACATGGT |  |
| qBnCCoAOMT1-F | ATCAAGCTCGTTAACGCCAAG | qRT-PCR for *BnCCoAOMT1* |
| qBnCCoAOMT1-R | GTTCCATGGTTCTTCTCGTCAGC |  |
| qBnCCR1-F | TGTTACGGCAAGATGGTGGCG | qRT-PCR for *BnCCR1* |
| qBnCCR1-R | AACATCCACATAAGCCTGAGT |  |
| qBnCOMT-F | AGGCATCAACTTTGATCTCCC | qRT-PCR for *BnCOMT* |
| qCOMT-R | TGGCATATCCACTTCATGA |  |
| qBnFAH1-F | ACTTATGACCGAGCCGACATG | qRT-PCR for *BnFAH1* |
| qBnFAH1-R | GAACGCTGCCCGGTAAGTTA |  |
| qBnPAL1-F | CAAGTCTACACGTACGCGGA | qRT-PCR for *BnPAL1* |
| qBnPAL1-R | TCAGGAAGCACCGCCTTGA |  |
| qBnPAL2-F | GCGATCATGGAGCACATCCTC | qRT-PCR for *BnPAL2* |
| qBnPAL2-R | GAGTTGATCTCACGCTCGAT |  |
| qBnCCR2-F | GGAGCCAAGTTCGTGATCGACG | qRT-PCR for *BnCCR2* |
| qBnCCR2-R | GCAATACCAATTCTTTGTGTT |  |
| qBnUBC9-F | TCCATCCGACAGCCCTTACTCT | qRT-PCR for *BnUBC9* |
| qBnUBC9-R | ACACTTTGGTCCTAAAAGCCACC |  |
| BnCCR2-F | ATGCCTGCCGACGGGAAAC | Clong of *BnaC.CCR2.b* |
| BnCCR2-R | TCTAGGATTCGATCGTGACGTTGT |  |
| qBnaC.CCR2.b-F | TTTACATGAACCCTAACCGTCA | qRT-PCR for *BnaC.CCR2.b* |
| qBnaC.CCR2.b-R | TAAGGTTAGCATAGGTCTTGGC |  |
| 35S-3 | TCCCACTATCCTTCGCAAG | Positive transformants identification |

**Table S2 Agronomic traits of transgenic lines in the T_2_ generation.**

| Line | J9712 | OE-1 | OE-2 | OE-5 | OE-6 |
| --- | --- | --- | --- | --- | --- |
| Plant height  （cm） | 147.9±12.4 | 146.1±8.2 | 147.6±11.5 | 151.0±14.6 | 138.8±21.2 |
| Main inflorescence lenght  （cm） | 59.8±17.4 | 67.3±9.4 | 67.8±11.9 | 60.6±12.4 | 60.5±5.8 |
| Effective branch height  （cm） | 35.8±5.5 | 32±4.7 | 30.3±4.7 | 36.0±8.5 | 34.5±5.6 |
| Effective branch number | 11±1 | 9±2 | 9±1 | 12±3 | 10±2 |
| Main inflorescence silique number | 60±6 | 62±14 | 69 ±20 | 69 ±9 | 54 ±14 |
| Silique number per plant | 562 ±97 | 584±69 | 504±75 | 701 ±77 | 633 ±91 |
| Silique lengh  （mm） | 79.4±2.6 | 79.6±3.6 | 80.4±3.1 | 81.7±4.0 | 79.1±3.9 |
| Silique seed number | 20 ±1 | 21±2 | 21 ±2 | 19 ±1 | 19 ±1 |
| Thousand-seed weight  （g） | 4.4±0.3 | 4.7±0.6 | 4.4±0.5 | 4.5±0.6 | 4.6±0.5 |
| Oil content (%) | 41.9±2.0 | 42.2±2.1 | 40.6±0.8 | 40.4±2.4 | 40.7±2.8 |

**
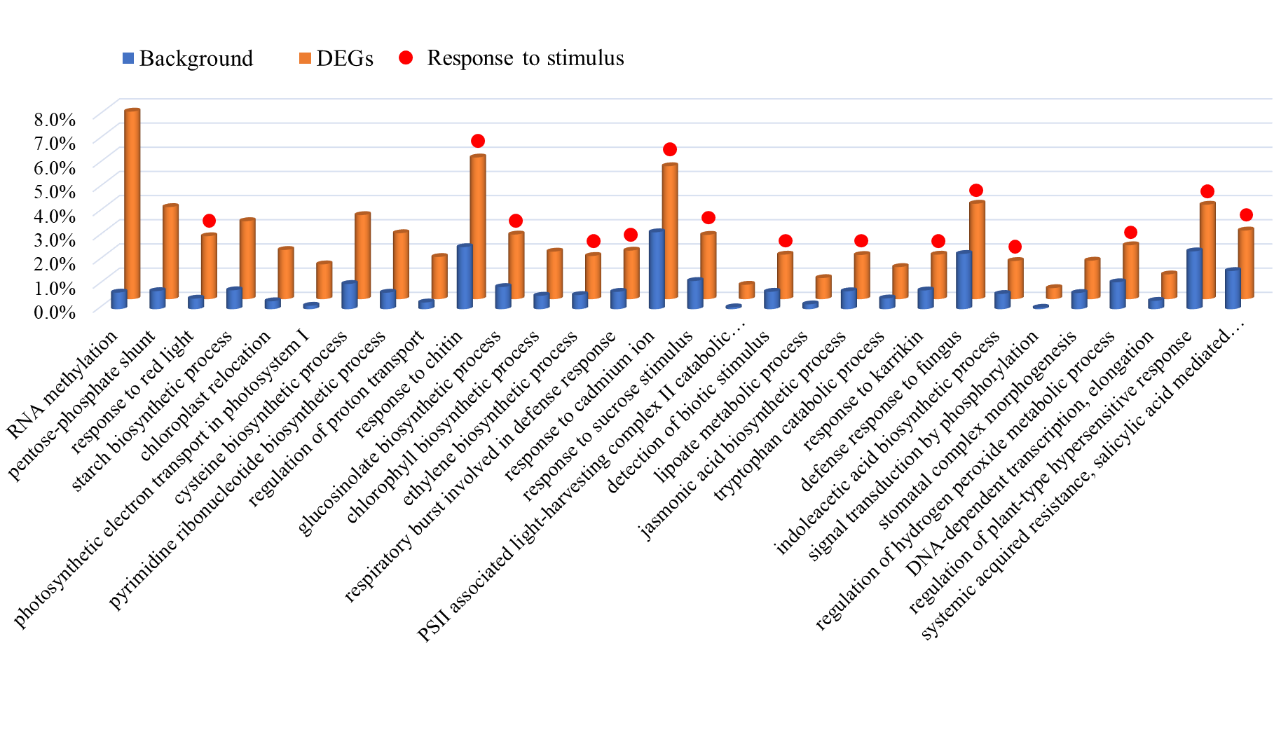
Figure S1 Biological process categorization of the upregulated genes at 24 hpi in the *Sclerotinia*-susceptible line (J902) based on GO enrichment analysis.** The y-axis is the percentage of genes mapped by the term, representing the abundance of the GO term. The percentage for the input list is calculated by the number of genes mapped to the GO term divided by the number of all genes in the input list. The same calculation was applied to the reference list (background) to generate its percentage. The top 30 enriched GO terms are shown.
